# Sex differences in Sjögren’s syndrome: a comprehensive review of immune mechanisms

**DOI:** 10.1186/s13293-015-0037-7

**Published:** 2015-11-03

**Authors:** Jessica E. Brandt, Roberta Priori, Guido Valesini, DeLisa Fairweather

**Affiliations:** Department of Environmental Health Sciences, Johns Hopkins University, Bloomberg School of Public Health, 615 N. Wolfe Street, Baltimore, MD 21205 USA; Reumatologia, Dipartimento di Medicina Interna e Specialita Mediche, Sapienza Universita di Roma, 00161 Rome, Italy; Department of Cardiovascular Diseases, Mayo Clinic, 4500 San Pablo Road, Jacksonville, FL 32224 USA

**Keywords:** Sjögren’s syndrome, Sex differences, Autoimmune disease, Inflammation, Autoantibodies, Lymphoma

## Abstract

Autoimmune diseases (ADs) are estimated to affect between 5 and 8 % of the US population, and approximately 80 % of these patients are women. Sjögren’s syndrome (SS) is an AD that occurs predominately in women over men (16:1). The hallmark characteristic of SS is diminished secretory production from the primary exocrine gland and the lacrimal or salivary glands resulting in symptoms of dry eye and mouth. The disease is believed to be mediated by an inflammatory and autoantibody response directed against salivary and lacrimal gland tissues. This review will examine the literature on sex differences in the immune response of patients and animal models of Sjögren’s syndrome in order to gain a better understanding of disease pathogenesis.

## Review

### Introduction

Autoimmune diseases (ADs) are estimated to affect between 5 and 8 % of the US population or 14.7 to 23.5 million people [1, 2]. Strikingly, around 80 % of those affected are women [1]. ADs with a recognized sex difference in incidence in women compared to men include multiple sclerosis (2:1), autoimmune hepatitis (4:1), Graves’ disease (7:1), and Hashimoto’s thyroiditis (19:1) [3, 4]. A female to male predominance also occurs for the rheumatic autoimmune diseases, dermatomyositis (2:1), rheumatoid arthritis (RA) (3:1), systemic lupus erythematosus (SLE) (7:1), systemic sclerosis (12:1), and Sjögren’s syndrome (SS) (16:1) [4–7]. ADs that occur more frequently in men compared to women include type 1 diabetes (1.2:1), idiopathic pulmonary fibrosis (11:7), and myocarditis (2:1) [4, 8]. Distinct immunopathologic differences between male and female predominant ADs suggest that sex hormone regulation of inflammation may provide clues to the pathogenesis of disease. Because inflammation and autoantibody responses are directly influenced by sex hormones, sex hormones most likely influence immune-mediated tissue damage triggered by agents like infections and chemicals in the context of genetic predisposition. This review will explore the literature on sex differences in the immune response of patient and animal models of SS in order to gain a better understanding of disease pathogenesis, with a focus on studies and review articles that have been published for SS.

Sjögren’s syndrome has an estimated prevalence of 0.5–4.8 %, affecting approximately 1.5 to 4 million people in the USA based on a total population of 300 million [9, 10], making it by some estimates the second most common autoimmune disease affecting women after Hashimoto’s thyroiditis [4, 11]. Because no prevalence studies have been performed in the USA, population-based incidence data from Olmsted County, Minnesota and prevalence data from several international studies have been used to infer SS prevalence in the US [12]. This study found a prevalence ranging from 0.2 to 1.4 %, placing SS second to RA among rheumatic diseases [12].

The hallmark characteristic of SS is diminished secretory production from the primary exocrine glands, the lacrimal (involved in tear production) and/or the salivary glands. As a result, dry eye (keratoconjunctivitis sicca) and/or dry mouth (xerostomia) are among the most commonly reported symptoms of SS. Aside from the exocrine targets, SS also affects the lungs, kidneys, thyroid, muscle, and skin and manifests with symptoms of fatigue, pain, depression, cutaneous lesions, and mild arthritis. Because SS symptoms can often be subtle, intermittent, and/or nonspecific, it has been estimated that symptoms can be present for 6 to 7 years before a diagnosis is made, dramatically reducing quality of life [13]. Historically, if the diagnosis is made in the absence of another AD, it has been classified as primary Sjögren’s syndrome (pSS), but SS is commonly associated with other ADs like rheumatoid arthritis, Raynaud’s phenomenon, and thyroiditis [14]. When patients present with a potentially associated disease (like another well-defined connective tissue disease), the disease has been termed secondary Sjögren’s syndrome (sSS) [15, 16]. More recently, the American College of Rheumatology provided a new classification criterion for SS suggesting that SS that occurs as a single entity should be called “isolated SS” and SS associated with other ADs be termed “associated SS” [17]. Additionally, they recommended that all those who fulfill the classification criteria should be diagnosed with SS regardless of whether they have another AD [17].

Autoimmune diseases are chronic inflammatory conditions where memory-specific T and B cells and antibodies are directed against self-antigens. Autoimmunity occurs frequently in undiseased individuals, but pathology or overt autoimmune disease generally develops when multiple or high titer autoantibodies are directed against a target tissue [8]. For this reason, a component of the classification criteria for most autoimmune diseases is based on the presence of particular autoantibodies in the sera. Pathogenic immune complexes (ICs) form when autoantibodies bind self-antigen and activate the complement cascade. Deposition of ICs often damages host tissues by causing direct cytotoxicity to host cells and recruiting inflammation to the damaged site [7, 18].

In addition to dry eyes and mouth, for a positive SS classification, patients may present with exocrine gland lymphocyte infiltration and/or autoantibodies against Ro (also called Sjögren’s syndrome-associated antigen-A/SSA) and/or La (SSB) (Table 1) [15, 17]. Ro and La were named from the first two letters of the surname of the donors who provided the reactive serum and are cytoplasmic and nuclear ribonucleoproteins (i.e., anti-nuclear antibodies (ANA)) found in all cells [19]. An overriding question remains as to why autoantibodies and ICs target the lacrimal and salivary glands when these antigens are present in every cell. Rheumatoid factor (RF), which are autoantibodies directed against the Fc portion of IgM, IgG, or IgA antibodies, are often found in autoimmune rheumatic diseases like SS, RA, and SLE. RF autoantibodies occur in 60–80 % of SS patients [20, 21] and have been found to predict the severity of disease in patients indicating that RF may contribute to the pathogenesis of disease and suggesting an important role for IC deposition in this process (Table 2) [20]. RF bound to other autoantibodies promotes IC formation and increases the potential for deposition. RF functions include enhancing normal immune responses, particularly against infections; promoting complement fixation, antigen presentation, and the avidity of IgG; and improving clearance of ICs by macrophages [20, 21]. These roles help clear infections but can also drive autoimmune disease. Plasma B cells within the salivary glands of SS patients have been found to produce Ro/SSA and La/SSB autoantibodies [22]. The inflammatory infiltrate in SS patients includes T and B cells, dendritic cells (DCs), and macrophages, while immune-mediated damage is believed to be driven by IC deposition, apoptosis, remodeling, and fibrosis [19].

**Table 1:** Revised European-American classification criteria for Sjögren’s syndrome (SS) modified from [15, 17]. Note that not all criteria are required for classification of SS

• Salivary gland inflammation confirmed by histology
• Autoantibodies against Ro/SSA and/or La/SSB
• Ocular signs and symptoms of dry eye
• Oral signs and symptoms of dry mouth including reduced salivary flow (≤1.5 mL in 15 min)

**Table 2:** Factors suggesting autoantibodies and ICs involved in the pathogenesis of Sjögren’s syndrome (SS)

• Female preponderance (i.e., estrogen’s central role in increasing autoantibodies)
• Women respond to infection, vaccination, and trauma with more antibodies compared to men
• Autoantibodies (i.e., Ro/SSA and La/SSB) part of SS classification criteria
• B/plasma cells in salivary glands of SS patients release Ro/SSA and La/SSB, which are ANA-type autoantibodies
• B cell hyperactivity (i.e., hypergammaglobulinemia) found in SS patients
• Pregnancy increases the risk for SS in young women; estrogen with prolactin increases Ro/SSA and La/SSB autoantibodies
• Some studies report more autoantibodies in women with SS compared to men
• Rheumatoid factor (RF), which forms ICs, present in 60–80 % of SS patients
• RF predicts severity of SS
• Elevated IgA in SS patients binds RF to form ICs and damage salivary glands
• Genetic predisposition in SS patients: low copy number of the IgG receptor FcγRIIIb gene *FCGR3B* reduces clearance of ICs
• B cells are activated by TLRs and BAFF on their surface; TLRs and BAFF correlate with autoantibody levels in SS patients
• IC deposition in salivary glands activates TLRs on epithelial cells generating the “IFN signature” that is associated with SS pathology
• Viral infections suspected in “triggering” SS activate TLRs to produce “IFN signature” as well as strongly inducing IC formation
• ICs known to produce tissue pathology by increasing inflammation, fibrosis, and apoptosis—endpoint characteristic of SS
• SS in women is associated with other ADs that occur predominantly in women like RA, thyroiditis, and Raynaud’s phenomenon—diseases where autoantibodies and ICs are believed to promote disease pathology

#### Immunopathogenesis of SS

A number of recent reviews describe the current understanding of the pathogenesis of SS [11, 23–25]. However, they do not specifically address the role of sex differences in disease pathogenesis, which is the purpose of this review. In general, recent reviews emphasize the importance of B cell activation and an “interferon signature” in the pathogenesis of disease, which has resulted in SS being called “lupus of the mucosa” by some [11]. Genetic polymorphisms in genes associated with activation of type I (α and β) and type II (γ) interferons (IFNs) (like IRF5 and STAT4) have been found to be associated with the development of SS [11, 25–27]. By microarray, 19 SS patients (all post-menopausal women) had an elevated IFN signature in peripheral blood compared to 10 healthy controls (5 men and 5 premenopausal women) [28]. The innate immune response appears to play a major role in the disease process. Toll-like receptors (TLRs) that are associated with viral infections and that upregulate NFκB and IFNs, like TLR3, TLR4, TLR7, and TLR9, are elevated in SS patients [25, 29]. Salivary gland epithelial cells from SS patients express not only TLR4 but also costimulatory molecules like CD86 indicating that they may play a role in activating the innate immune response [11, 29]. This may be especially important after damage to the salivary gland since TLR4 is activated not only by infections but also by damaged self-tissue (DAMPs).

B cell hyperactivity is another hallmark of SS. Not only does activation of B cells result in hypergammaglobulinemia and autoantibodies against Ro/SSA and La/SSB but also B cell activation via TLRs and B cell activating factor (BAFF) on their surface increases IFNs [11, 24]. BAFF is further elevated by IFNs in a positive feedback loop. Elevated BAFF levels in SS patients were found to correlate with their autoantibody levels [30]. Elevated IgA that binds RF to form ICs is a common finding during SS and these IgA-RF ICs are strongly associated with an abnormal SG biopsy in patients [24, 31, 32]. IC deposition in SLE and RA is known to activate TLRs leading to increased IFNs, and it has been hypothesized that autoantibody/ICs could be important in the pathogenesis of SS [11, 24]. A summary of data suggesting a potential role for autoantibodies and ICs in the pathogenesis of SS are listed in Table 2.

Viral infections, which strongly activate ICs and drive IFN responses via TLRs, have long been implicated as a trigger for autoimmune diseases like SLE, RA, and SS [11, 23–25]. But so far, no associations have been found between viral candidates like Epstein-Barr virus, hepatitis C virus or retroviruses, and SS patients [11]. The long time frame between initiation of disease and clinical diagnosis makes it difficult to determine trigger events like viral infection, which may have resolved months or years previously. Viral infections that were cleared months or years earlier are not likely to account for the IFN signature in SS because the immune response returns to normal after viral clearance, and persistent viruses frequently “persist” because they do not induce strong IFN responses. However, recent or reactivated viral infections would promote IFNs.

#### Sex differences in the immune response

An unresolved question is why most autoimmune diseases occur more frequently in women than men. It is well known that women respond to infection, vaccination, and trauma with increased antibody production [33, 34]. Although increased antibody levels protect women from infections, they also appear to increase the risk of developing ADs. Estrogen activates B cells resulting in increased levels of antibodies and autoantibodies, while androgens decrease B cell maturation, reduce B cell synthesis of antibody, and suppress autoantibody production in humans [35–37]. Microarray and other molecular tools have revealed in recent years how the immune response under normal and pathologic conditions is extensively regulated by sex hormones. Sex steroid hormone receptors such as the estrogen receptor (ER)-α, ER-β, androgen receptor, and aromatase, the enzyme that converts androgens to estrogens, are expressed on the cell surface and/or intracellularly in immune cells. Likewise, cytokine receptors like interleukin (IL)-1 receptor (IL-1R) are found on classic hormone-producing tissues, indicating immune regulation of sex hormones [38].

In cell culture studies and animal models, estrogen has been shown to induce differentiation of DCs, stimulate T cell proliferation, and increase T helper (Th)2 responses, regulatory T cells (Treg), IL-4-driven alternatively activated M2 macrophages, and the regulatory cytokines IL-4, IL-10, and transforming growth factor (TGF)β [7, 35, 39–42]. Estrogen has also been shown to inhibit innate TLR responses and NF-κB thereby reducing IFNγ production from immune cells [37, 40, 43, 44]. Intriguingly, autoantibodies against ERα have been found to interfere with T cell homeostasis and to be significantly associated with disease activity in SLE patients [45]. That the immune response would generate autoantibodies directed to ERα suggests that estrogen is contributing to the disease process in rheumatic ADs.

Far less research has been conducted on the effect of androgens on immune cell function, but in general, androgens drive IFNγ-associated Th1 responses [46, 47]. One complication in understanding the role of sex hormones on immune cells is that most studies do not interpret data in the context of sex. For example, the sex of cells used in culture experiments is seldom reported [48]. Additionally, effects measured by testosterone may be due to testosterone or estrogen because of aromatase conversion. In support of testosterone driving Th1 responses and estrogen driving Th2 responses, Dube et al. found in a retrospective cohort study of 15,357 adults that more men than women were hospitalized for autoimmune diseases associated with Th1-type pathology, while more women than men had autoimmune diseases associated with a Th2-type response [49]. In particular, Th2-associated rheumatic autoimmune diseases occurred more frequently in women than men (adjusted hazards ratio 2.5, 95 % confidence intervals 1.6–3.9) [49]. Because Th1 (IFNγ) and Th2 (IL-4) responses cross-regulate each other transcriptionally, the effect of estrogen or testosterone in skewing Th responses may be exaggerated. Thus, estrogen’s central role in driving a Th2-type skewed immune response that activates B cells and elevates autoantibody and IC levels could be a contributing factor in the increased risk for SS in women after triggers like infection.

#### Risk factors

##### Pregnancy

To date, there have been just two retrospective case-control studies specifically investigating risk factors for developing SS; the first study aimed to identify perinatal influences associated with developing SS as an adult, and the second focuses on familial, menstrual, and pregnancy history as factors contributing to increased risk [50, 51]. By means of a 32 case (28 women to 4 men) and 159 control (139 women to 20 men) case-control study, Mostafavi et al. noted significantly increased odds ratios (OR) for low maternal age (26.1 vs. 28.3 years of age, *p* = 0.04) and high infant birth weight (OR = 3.8, 95 % confidence intervals (CI): 1.3–11.7) as well as associations of SS with being the first-born child (OR = 2.7, 95 % CI: 1.1–6.8), maternal diseases during pregnancy (OR = 3.9, 95 % CI: 0.9-16.9), and paternal socioeconomic status as a manual worker (OR = 3.9, 95 % CI: 1.1-13.4) [50]. How perinatal “stressors” (i.e., infections, first child, low income) influence the immune response is not yet clear, but a good candidate includes epigenetic regulators like microRNAs [52]. Although this study included men and women, the data were not analyzed by sex.

In the second case-control study, Priori et al. evaluated 140 female SS patients (39 or 27.9 % were sSS) and 109 female controls [51]. Of the evaluated characteristics (consanguinity, smoking habits, history of blood transfusions, obstetric history, and family history of AD), a significant association was observed with positive first-degree relative history of AD (OR = 7.4, 95 % CI: 2.8–20.1) and in women with one or more pregnancies (OR = 2.2, 95 % CI: 1.1–4.2) or births (OR = 2.5, 95 % CI: 1.3–4.7) [51]. However, no association was found between the age at menarche or menopause and the risk of developing SS. More mothers, sisters, and daughters had ADs compared to male first-degree relatives, but this is not surprising because the ADs of first-degree relatives were ADs that occur predominantly in women [8, 51]. Interestingly, pregnancy and the presence of other ADs in patients (i.e., associated SS) appeared to act synergistically to increase the risk of developing SS. Patients with multiple ADs are likely to have more distinct types of autoantibodies, which predict risk for developing an AD [53]. It has been found that if three autoantibodies are present compared to just one type of autoantibody, an individual has a 60–80 % increased risk of developing an AD [54]. It should be noted however that SS patients can have successful pregnancies [55].

Additionally, during pregnancy, prolactin and estrogen levels rise. Several clinical and animal studies have found an association between elevated prolactin levels and SS [56–59]. Not only does estrogen increase prolactin levels but also estrogen and prolactin work together to increase autoantibody levels and Ro/SSA and La/SSB autoantibodies in particular [56, 60, 61]. However, IL-1β and IL-6 also increase prolactin levels, and so, it is not yet clear whether elevated prolactin drives disease pathogenesis or occurs as a result of pathology [61]. Regardless, once prolactin is elevated, it is able to directly alter immune function via prolactin receptors expressed on immune cells like mast cells, macrophages, and T and B cells resulting in elevated autoantibodies [61]. Thus, pregnancy and the presence of other ADs increase the risk for developing SS. Two peaks for the appearance of SS have been reported, one during child-bearing years around age 30 and far more frequently shortly after menopause around age 55 [62]. Thus, prolactin may work together with high estrogen levels to increase the risk for SS in premenopausal women.

##### Menopause

**Fig. 1:**
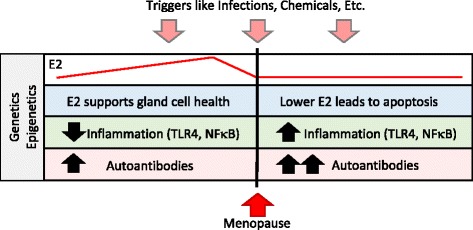
Possible role of estrogen in promoting Sjögren’s syndrome. In the context of genetic, epigenetic, and environmental influences like infections and chemicals, the rapid decline in estrogen (E2) levels prior to menopause leads to reduced glandular cell health. Death of glandular cells via apoptosis/necrosis provides self-antigens like nuclear antigens for presentation to the immune system to promote autoimmune disease. At the same time, the protective effects of higher estrogen levels on inflammation disappear allowing increased activation of innate immune pathways like toll-like receptor 4 (TLR4) and NFκB. In contrast, low levels of estrogen continue to increase the level and different types of autoantibodies with age

Female predominance and disease onset following a major stressful event and/or following menopause in SS patients suggest that the endocrine system and sex hormones are involved in the pathogenesis of disease [63]. Menopause is defined as the final menstrual period without another menstrual period for 12 months and occurs in Western cultures at 49–52 years of age [64]. Large decreases in estrogen (i.e., estradiol) occur in the last 6 months before menopause and thereafter, while testosterone gradually decreases with age in both sexes. Understanding how the menopausal transition affects inflammation is complicated by changes in the immune response that occur with aging. Autoantibodies continually increase with age in women [65–67]. Importantly, lower doses of estrogen present after menopause are still able to promote B cell proliferation and autoantibody production following menopause in women (Fig. 1). However, lower estrogen levels may reduce the protective, anti-inflammatory effects of estrogen (i.e., estrogen increases Treg and regulatory macrophages and inhibits proinflammatory NFκB) that were present prior to menopause and allow increased activation of TLR on innate immune cells resulting in elevated proinflammatory cytokines like TNF, IL-1β, and IFNs [7] (Fig. 1).

That most women develop SS after menopause (age 55 or older) suggests that estrogen in general protects against the development of SS [62, 68]. Alternatively, it may just take more “time” to produce multiple autoantibodies (Fig. 1). Evidence to support a protective role for estrogen in SS can be found in a recent study in mice where ovariectomy of adult females (modeling menopause) in a SS model significantly increased inflammation in the lacrimal (tear) gland [69]. Estrogen replacement of ovariectomized mice in these studies reversed the effect by reducing T and B cell infiltration, suggesting that estrogen reduces tear gland inflammation during SS. Note that recruitment of B cells to tissues involves different immune signaling pathways than activating B cells to produce antibody/autoantibodies, so that estrogen would be expected to decrease inflammation but increase antibodies from B cells at the same time. Ovariectomy of wild type female mice has also been reported to cause SS-like disease with apoptosis of salivary gland epithelial cells, indicating that estrogen is needed for salivary gland cell survival [70]. Note that apoptosis provides an important source of self-antigen leading to the generation of self-reactive T and B cells and autoantibodies [18, 71]. Thus, reduced levels of estrogen with menopause could decrease the protective effect of estrogen by increasing inflammation and by reducing its proliferative effects on glandular cells leading to increased apoptosis (Fig. 1). In support of this idea, low salivary estrogen levels have been found to correlate with a feeling of dry mouth in healthy menopausal women [72] and low circulating estrogen levels with ocular dryness in SS patients [73]. Salivary gland epithelial cells express ERα and ERβ, and these cells from SS patients have been found to have reduced responsiveness to estrogen (i.e., 17β-estradiol) [74, 75], a factor that may be linked to the menopausal transition. Additionally, aromatase deficient mice, which should prevent the conversion of androgens to estrogen resulting in lower estrogen, develop a SS-like disease with increased inflammation of exocrine glands [76].

However, the role of sex hormones in SS is not straight-forward. Oral dryness in SS patients has also been linked to low circulating androgen levels [73]. Recall that androgen as well as estrogen levels drop in women during and after menopause. Circulating dehydroepiandrosterone (DHEA), an adrenal prophormone that can be metabolized to estrogen and testosterone, is around 50 % lower in SS patients than age- and sex-matched controls [77]. Salivary glands from SS patients have also been found to have reduced levels of DHEA [78]. Similar to the protective role of estrogen, DHEA is needed for renewal of acinar cells of the labial salivary glands and apoptosis of salivary gland cells can be reversed by treatment of mice or patients with androgens [73, 79, 80]. Thus, both estrogen and androgens are needed for normal exocrine gland function. The reduction in circulating sex hormone levels leading to increased apoptosis of exocrine gland cells combined with the ability of estrogen, even at low doses, to promote autoantibody formation could increase the risk in women of developing SS (Fig. 1). Thus, these data support the idea that estrogen protects against SS prior to menopause but contributes to increased pathology after menopause by increasing inflammation, autoantibody levels, and death of tear duct and salivary gland cells resulting in dry eye and mouth.

##### Immunogenetics

There are a number of genes linked to increased risk for developing SS. As for other ADs, genetic and epigenetic associations with SS center on alterations in genes that regulate immune function [81, 82]. Low copy number of the IgG receptor FcγRIIIb gene *FCGR3B*, which facilitates clearance of ICs, was associated with risk for developing isolated SS in a cross-sectional study of 174 SS patients compared to 162 controls (OR 2.3, 95 % CI 1.3–3.9, *p* = 0.003) [83]. Although 90 % of the SS patients were female, while only 53 % of the controls were female; no relationship was observed between *FCGR3B* copy number and sex [83]. But in order to draw conclusions about sex differences in polymorphisms in this gene, a similar percentage of female patients and controls should be examined.

Polymorphisms in *IRF5* and *STAT4* genes have been associated with increased risk of isolated SS as well as other ADs including SLE, Hashimoto’s thyroiditis, primary biliary cirrhosis, Graves’ disease, scleroderma, and RA [11, 25–27, 84–86]. These genes promote Th1-type immune responses by increasing type I (IFNα/β) and type II (IFNγ) IFNs. Type I mRNA (i.e., IFNα) is upregulated in the salivary glands and peripheral blood cells of SS patients [84, 87, 88], but circulating IFNs were not found to correlate with mRNA levels [84]. However, IL-12, a cytokine that activates STAT4 leading to Th1 responses, has been found to be elevated in the circulation of SS patients compared to controls [89]. Although it has become clear more recently that sex hormones influence Th1 vs. Th2 development, these studies did not examine whether sex differences exist in *IRF5* or *STAT4* polymorphisms in SS patients and in some cases did not list the sex of the patients or controls in the study [26, 27, 84].

MicroRNAs (miRNAs) are small non-coding RNAs that post-transcriptionally regulate gene expression. Expression of miR-146a has been found to be increased in peripheral blood cells and salivary glands of mice and SS patients compared to controls [90, 91]. miR-146a is upregulated in macrophages following activation of TLR4 by lipopolysaccharide and is thought to inhibit products of TLR4 signaling like IL-1β and IL-18 [92]. However, IL-1β and IL-18 are elevated in the circulation and salivary glands of SS patients and mice compared to controls [93, 94]. Additionally, polymorphisms in IL-1β and IL-1R antagonist (IL-1RA) increase susceptibility to SS [95, 96]. Although IL-1β and IL-18 (originally named IFNγ-inducing factor) are associated with proinflammatory responses and IL-18 increases Th1 responses, TLR4-induced IL-1β and IL-18 are also produced from alternatively activated, Th2-associated M2 macrophages and mast cells [6, 8]. Thus, upregulation of miR-146a in SS patients may be an attempt to regulate the expression of IL-1β and IL-18. Although to our knowledge, no one has studied whether sex differences in miR-146a exist, as mentioned earlier, estrogen is known to inhibit TLR4 signaling. Thus, lower estrogen during and following menopause would be expected to allow elevation of this pathway in the context of TLR4 triggers like infection. Studies on the role of sex hormones in this pathway in SS patients or animal models are warranted.

Lastly, regulatory T cells (Treg) that express Foxp3 are known to be under epigenetic control. Treg, which are able to downregulate Th1-, Th2-, and Th17-type immune responses, are increased in frequency in salivary glands and the peripheral blood of SS patients compared to age and sex-matched controls [97, 98]. Foxp3^+^ Treg levels were found to positively correlate with the severity of disease in SS patients including infiltrating monocyte/macrophages, DCs, and serum complement C4 levels [97]. This may seem somewhat surprising since elevated Treg should inhibit inflammation. Although Foxp3^+^ Treg may be attempting to regulate the immune response in SS patients [98], their numbers can also be increased by estrogen, Th2-type immune responses, and TLR4 signaling [7, 44]. What is generally missing from the study of genes that increase the risk for SS is an assessment of whether sex differences exist.

#### Sex differences in serological markers

A component of the classification criteria for SS is based on the presence of particular autoantibodies in the sera (Table 1). Sjögren’s syndrome is commonly associated with serological changes including hypergammaglobulinemia and the presence of paraproteins, monoclonal immunoglobulin light chains that are produced in excess. In addition, a diverse autoantibody profile of RF, ANA, Ro/SSA, and/or La/SSB is observed [99]. There are a number of studies that have considered whether sex differences exist in the presence of autoantibodies between men and women with SS (Table 3) [100–109]. Six of these studies, totaling 1570 individuals with SS, found autoantibodies were increased in women compared to men (Table 3). In contrast, 3 studies of a total of 950 SS patients found no sex difference in autoantibodies, and one study of 549 patients found increased autoantibodies in men (Table 3). The largest single study, which compared 938 women to 73 men with pSS, found that more women were positive for ANA than men [109]. Recall that Ro/SSA and La/SSB are types of ANA autoantibodies. One of the obstacles in drawing conclusions from studies with fewer individuals is that it may be difficult to obtain significant differences between men and women because of the small number of men in the study. Additionally, men who are recruited to studies may have unusually high autoantibody levels because the presence of autoantibodies is part of the classification criteria for SS, thereby reducing the potential for sex differences in autoantibodies to be present.

**Table 3:** Sex differences in serology and extraglandular manifestations during Sjögren’s syndrome

Study (reference) site	Classification criteria	*n* (F/M)	Serologic differences F > M	Extraglandular manifestations F > M
1986 Molina [100] USA	N/A	105 (69/36)	RF, Ro/SSA, La/SSB	No differences
1995 Anaya [102] USA	European [103]	39 (26/13)	No differences	No differences
1997 Drosos [103] Greece	European [103]	42 (30/12)	ANA, Ro/SSA	Raynaud’s phenomenon, polyarthritis
1999 Brennan [104] USA	European [103]	42 (28/14)	ANA, Ro/SSA	Fatigue
1999 Saito [113] Japan	American-European [13]	129 (117/12)	Not performed	Inflammation, sialectasis
2000 Cervera [101] Spain	European [103]	223 (204/19)	RF, ANA	Raynaud’s phenomenon, cutaneous vasculitis, polyarthritis
2004 Diaz-Lopez [105] Spain	European [103]	549 (521/28)	M > F: RF, ANA, IgA	Fibromyalgia, thyroiditis
2007 Goeb [106] France	American-European [13]	148 (137/11)	ANA, Ro/SSA, La/SSB	Raynaud’s phenomenon
2008 Horvath [107] Hungary	American-European [13]	492 (432/60)	No differences	Raynaud’s phenomenon, thyroiditis
2008 Gondran [108] France	American-European [13]	419 (377/42)	No differences	Lymphopenia, leucopenia
2008 Ramos-Casals [109] Spain	European [103]	1,010 (938/73)	ANA	Raynaud’s phenomenon, thyroiditis

In summary, autoantibodies were found to be elevated in women with isolated SS compared to men in a greater number of studies. In general, women were more frequently positive for ANA (five studies), Ro/SSA (four studies), and La/SSB (two studies) (Table 3) [100, 101, 103, 104, 106, 107, 109]. However, two large studies did not confirm this assessment [105, 108]. All studies but two reported no sex difference for RF. There are several possible explanations for the inconsistencies that occurred between studies. Some studies used the European classification criteria [110], while others used the American-European criteria [15]. Most studies had a female to male ratio of 9:1 but Diaz-Lopez et al. studied a population with a ratio of 18:1 [105] and the ratio was 13:1 for the Ramos-Casals et al. study [109], which may have altered the outcome. Most studies examined patients around 55 years of age, but a few studies examined a more elderly (i.e., 65 years) or younger (i.e., 45 years) population. It is also likely that differences exist in the ability of different ELISAs to detect autoantibodies, which would alter detection rates. Additionally, many different ethnic groups are represented in these studies, which may differ in susceptibility to SS. In spite of these differences, a number of studies have found that SS-associated autoantibodies occur more frequently in women with isolated SS. However, more research is needed in this area to determine whether sex differences in autoantibody levels exist and if so, how this difference impacts the pathogenesis and diagnosis of disease.

#### Sex differences in clinical manifestations

Isolated SS in men is rare. Though no study has been designed to measure the prevalence of isolated SS in men, the relatively low number of males in the published literature to date supports this statement (Table 3) [100, 101, 103, 104, 106, 107, 109, 111]. Paradoxically, in spite of the overwhelming prevalence of the disorder in women, the first patient described to have primary Sjögren’s syndrome by Mikulicz in 1888 was a 42-year-old male farmer with bilateral parotid and lacrimal gland enlargement [112].

There are a number of clinical SS studies focused on differences in extraglandular disease expression between the sexes (Table 3) [100–109, 113, 114]. Two studies did not observe sex differences in extraglandular manifestations [100, 102]. However, the patients in the Anaya et al. study (the age of patients in the Molina et al. study was not described) were young (<40 years of age), which may account for the observation since tissue damage would be expected to take time to develop. In one of the largest cohorts published to date, where 73 of 1010 patients were male (7 %), a lower frequency of Raynaud’s phenomenon and thyroiditis was found in men compared to women (Table 4) [109]. This observation was confirmed in several other studies [101, 103, 106, 107]. Additionally, female SS patients show a higher frequency of depression [108], fibromyalgia/fatigue [104, 105], and thyroiditis [105, 109]—conditions known to occur more frequently in women than men [8, 115–117].

**Table 4:** Summary of sex hormone effects on Sjögren’s syndrome (SS)

• Estrogen activates B cells increasing autoantibodies and ICs, testosterone inhibits B cells decreasing autoantibodies and ICs
• Some studies report more autoantibodies in women with SS compared to men
• Exocrine gland inflammation is elevated in women with SS compared to men
• SS in women is associated with other ADs that occur predominantly in women like RA, thyroiditis, and Raynaud’s phenomenon
• Lymphoma occurs more frequently in men with SS
• Most cases of SS occur in women following menopause indicating that, in general, estrogen protects against SS prior to menopause
• Prolactin released during pregnancy may increase premenopausal SS cases
• Prolactin acts synergistically with estrogen to increase Ro/SSA and La/SSB
• Low estrogen levels after menopause continue to elevate autoantibodies
• Estrogen protects against SS-like disease in animal models
• The androgen DHEA protects against SS in patients
• Estrogen and DHEA is decreased following menopause when most SS cases occur
• Low estrogen levels correlate with dry mouth and ocular dryness

Raynaud’s phenomenon occurs most frequently with connective tissue autoimmune diseases (96 %) like mixed connective tissue disease (86 %), SLE (31 %), RA (22 %), and SS (13 %) [117]. The main pathology of Raynaud’s phenomenon is associated with dysregulation of autonomic and small sensitive nerve fibers resulting in abnormal, long-lasting vasoconstriction of peripheral small vessels [116, 117]. ANA autoantibodies are found in patients with Raynaud’s phenomenon and are believed to be involved in the pathogenesis of disease [117]. Sex hormones are also thought to play a role in the pathogenesis of disease, with the incidence of Raynaud-like vasospastic reactions increased during the pre-ovulatory period and with estrogen administration [118].

In contrast, a disease that is dominant in men over women with isolated SS is lymphoma (Table 4). Anaya et al. reported the prevalence of malignant lymphoma in men with isolated SS to be 15.4 % [102], higher than the 4–9 % commonly reported [111, 119–121]. The observation of a higher risk of lymphoma in men has been recently confirmed in a large group of patients with isolated SS from Italy where cumulative demographic, clinical, serologic, histological, and therapeutic data of 1115 (1067 women is to 48 men) isolated SS patients were retrospectively evaluated. Fifty cases of non-Hodgkin’s lymphoma were documented with an overall prevalence of 4.5 % and male sex was established as an independent risk factor [111]. An additional three studies demonstrated a slightly higher but not significant prevalence of lymphoma in men compared to women with isolated SS [106, 108, 122]. According to Gondran et al., lymphoma development occurs earlier in males than females following SS diagnosis [108]. These reports correspond with the observation that isolated SS mortality in men is three times that in women, at least in Eastern populations where both the onset and diagnosis of SS occurs later for men [104, 105, 107, 108, 123]. Overall, the strength of the association between lymphoma development and SS seems to be stronger in men than women, even if the underlying autoimmune disease prevalence is, as expected, far higher in women than men [124, 125]. In general, lymphoma has been reported to occur more frequently in men than women [126]. Animal models have shown that T cell lymphomas are larger in male compared to female mice [127]. And finally, women taking oral contraceptives have been found to have a reduced risk of developing non-Hodgkin’s lymphoma by up to 50 % [128, 129]. These data suggest that sex hormone regulation of immunopathology determines sex differences in extraglandular presentations during SS.

#### Sex differences in SS immunopathology: evidence from animal models

Concerning the immunological expression of disease, most authors agree that whether according to histologic, sialographic, and/or immunologic assessment, SS is slightly less severe in men than in women (Tables 3 and 4) [100–103, 109, 113, 114]. A small study by Saito et al. found a lower prevalence of SS-related clinicopathologic and sialographic changes in men with SS than in women [113]. The mean stimulated parotid flow rate was higher in men than women while the prevalence of SS-related sialographic findings, such as globular and punctate sialectasis, and grade 4 cases based on labial salivary gland biopsy were lower in men compared to women with SS [113], suggesting men have less inflammation than women. Additionally, ocular objective tests seem to be less frequently altered in men [109]. Lastly, in a study comparing 377 women to 42 men, Gondran et al. found greater frequency of lymphopenia and leucopenia in women, but greater thrombopenia in men [108].

Consistent with clinical observations, animal models of SS have found that female mice have greater lacrimal and salivary gland inflammation than males in animal models of SS with a more predominant Th2 and Th17 response and more B cells [130–134]. An epidemiologic study found that hormone replacement therapy in post-menopausal women increased the risk of dry eye syndrome [135], suggesting that high doses of estrogen could increase inflammation. In contrast, ovariectomy of adult female mice in a SS model significantly increased inflammation in the lacrimal gland [69], indicating estrogen reduces inflammation. In support of this, estrogen replacement of ovariectomized mice reduced T and B cell infiltrations, indicating that estrogen reduces lacrimal gland inflammation during SS.

The clinical and mouse data appear to be contradictory, but there is a possible explanation. High doses of estrogen are known to have an opposite effect on inflammation than low doses [6, 8], and so, hormone replacement therapy may not exactly mimic pre-menopause conditions. Estrogen is known to decrease inflammation by increasing Treg, Th2 responses, and regulatory cytokines like IL-10 and TGFβ [7, 35, 39–42]. In contrast, high doses of estrogen can increase inflammation, like during pregnancy and with hormone replacement therapy. Estrogen, but not testosterone, is necessary for increased antibody and autoantibody levels and thereby participates in the development of ICs. This is true even for low doses of estrogen. IC deposition is a strong activator of complement, innate immune mechanisms like TLR4 and increases inflammation [6]. Thus, an explanation for the data is that estrogen in females is increasing inflammation via autoantibody/IC-mediated mechanisms. In other words, estrogen can increase inflammation through the antibody arm of the immune response rather than the classical immune cell-mediated arm. Thus, reduced estrogen with menopause decreases the regulatory, protective effects of estrogen on inflammation but should not reduce its ability to promote inflammation via autoantibodies and ICs.

Testosterone may also increase inflammation during SS. Post-menopausal women with isolated SS were found to have higher levels of testosterone that positively correlated with disease activity [136]. There is evidence to suggest that testosterone increases classic inflammation but not autoantibody/IC levels. Although far less research has been conducted on the effect of androgens on immune cell function, in general, androgens drive cell-mediated IFNγ-associated Th1 responses and inhibit antibody production [46, 47]. For this reason, men are less likely to meet the classification criteria for diagnosis of SS. Importantly, decreases in estrogen levels following menopause could decrease the protective effect of estrogen including its organ-promoting proliferative effects on glandular cells leading to increased apoptosis and disease (Fig. 1) [80]. That most cases of isolated SS occur shortly after menopause at around age 55–60 suggests that the sudden drop in estrogen and/or a shift in the estrogen to androgen ratio contribute to disease pathology [137]. In support of this idea, low salivary estrogen levels have been found to correlate with a feeling of dry mouth in healthy menopausal women [72].

Another theory proposed to explain the sex difference in SS suggests that lower estrogen levels in females after menopause reduce salivary gland-specific TGFβ production allowing increased inflammation. Microarray conducted on normal salivary glands from men and women without SS found that women had lower expression of TGFβ compared to men [138]. Wild type female mice with the TGFβ receptor inactivated developed salivary gland inflammation and an increased Th1-type immune response [139], suggesting that this mechanism could influence inflammation.

Not only is estrogen lower following menopause but also the adrenal pro-hormone DHEA, which is reduced by around 50 % in SS patients compared to healthy age- and sex-matched controls [77]. DHEA is important for the repair and renewal of acinar cells of the labial salivary glands and impaired levels of DHEA can lead to apoptosis of these cells [80]. DHEA has been used as a therapy in SS patients, where administration reduced symptoms of dry mouth [73]. Reduction in DHEA with menopause resulting in apoptosis could lead to upregulation of TLR and activation of the inflammasome (e.g., TLR4, caspase-1, IL-1β, IL-18, IFNs) leading to salivary gland inflammation. The inflammasome does appear to be activated during SS in humans and animal models [94, 140]. Genetic polymorphisms in IL-1β or IL-1 receptor antagonist (an inhibitor of IL-1R signaling) are believed to affect SS [95, 96], and IL-1 is one of the prominent cytokines detected in salivary glands of SS patients [141]. Additionally, IL-18, a component of the inflammasome, was found to be present in macrophages within inflammatory foci of the salivary gland of SS patients and circulating IL-18 levels were elevated in SS patients compared to controls [93]. Interestingly, serum IL-18 levels were strongly correlated with Ro/SSA and La/SSB autoantibodies. Although SS has traditionally been considered a Th1-driven immune response, this could be due to IL-18 rather than the classical IL-12-induced Th1 pathway because IL-18 strongly drives Th1 differentiation resulting in elevated levels of IFNs. Activation of the inflammasome results in a mixed Th1/Th2-type immune response [6–8]. Th2 responses are required for increased autoantibody levels. A mixed Th cytokine profile has been demonstrated in the salivary gland of patients where IL-4, IL-13, IL-17A, and IFNγ levels were found to be significantly higher in SS patients compared to controls [142, 143]. Additional support for the idea that Th2 responses could be important for SS comes from the finding that SS patients had significantly higher levels of circulating IL-13 compared to controls [144].

#### Microchimerism

Although this review has focused on sex differences in the immune response during Sjögren’s syndrome, the possibility that the increased incidence of disease in women could be due to microchimerism should also be mentioned. Microchimerism is the presence of non-host stem cells or their progeny in an individual at a low level. This occurs during pregnancy, in twins and with blood transfusions or transplantation [145–147]. Microchimerism can occur as early as 6 weeks into pregnancy. A wide variety of fetal cells have been detected in the maternal circulation including mesenchymal stem cells, hematopoietic progenitor cells, nucleated erythroblasts, CD34^+^ stem cells, and immune cells like monocytes, natural killer cells, T cells, and B cells [147]. This finding led to the hypothesis that microchimerism in women following pregnancy with sons may be involved in the pathogenesis of autoimmune diseases. This concept was also thought to account for sex differences in disease because mothers with non-host cells from their sons may be predisposed to develop autoimmune disease if they have the right genetic background (i.e., HLA susceptibility for example) [145]. The primary mechanism is thought to involve a graft-vs.-host (GVH) inflammatory response, which is the primary inflammatory concern following blood transfusions and transplantation including stem cell transplantation.

Initial evidence that microchimerism may be involved in SS comes from a number of reports of patients developing a SS-type disease following blood transfusion, stem cell transplantation, or during GVH disease [148–150]. Several studies have examined SS patients to determine whether microchimerism exists. Endo et al. compared women with SS to healthy control women who had given birth to at least one son and found evidence of male cells in the salivary gland biopsy of 11/20 patients compared to 1/8 controls [151]. A similar study by Kuroki et al. found that 10/28 women with SS and previous male pregnancies had evidence of microchimerism in their salivary gland while 0/10 control women did not [152]. All studies so far agree that if microchimerism exists, it is found in the salivary gland and not in peripheral blood cells. However, several other studies found no evidence of microchimerism in SS women who had given birth to at least one boy [153–155].

Although this is an interesting theory, there are a number of issues that need to be resolved. One is that GVH disease occurs suddenly and aggressively when foreign cells are introduced to the host, but SS peaks after menopause around 20 to 30 years after giving birth to a boy. So if GVH disease is a pathomechanism, why does it take so long to develop disease? Secondly, microchimerism is bi-directional meaning that boys also obtain cells from their mothers [145–147]; so why wouldn’t men also be predisposed to autoimmune/GVH disease by this mechanism? Thirdly, it is not uncommon for healthy individuals to develop microchimerism [145], so perhaps the associations found by Endo et al. and Kuoki et al. would diminish with an increased sample size. Additionally, there is some evidence that microchimerism may be immunoregulatory and reduce the risk of graft rejection if a microchimerism is present [147, 156, 157]. And finally, microchimerism is known to persist in immunodeficient individuals [146], so the possibility exists that microchimeric cells simply persist in patients with SS because their immune response is dysregulated. Thus, little clear evidence exists for the role of microchimerism in the pathogenesis of SS, and more research is needed to determine whether this mechanism could skew the prevalence of disease toward women.

## Conclusions

Although Sjögren’s syndrome can occur in women during child-bearing years, most cases of SS occur soon after menopause around age 55–60. Clinical and animal model evidence indicates that estrogen and androgens like DHEA promote gland cell survival and protect against exocrine gland inflammation; and these hormone levels decline at menopause. Even though estrogen levels drop significantly prior to menopause and androgens gradually decrease, low levels of estrogen continue to drive autoantibody diversity. Other female-dominate rheumatic ADs like SLE and RA increase the risk of developing SS and generate similar autoantibody profiles. Extraglandular manifestations of SS follow typical sex difference predominance with thyroiditis, Raynaud’s phenomenon, depression, and fibromyalgia occurring more frequently in women than men, while lymphoma occurs more frequently in men. Overall, estrogen may increase the incidence of SS in women by increasing autoantibody production, even following menopause, leading to IC deposition, tissue damage, TLR/ inflammasome activation, elevated IFNs, and exocrine gland dysfunction. Importantly, sex hormones most likely contribute to SS pathology in the context of genetic predisposition and environmental triggers like infections.
